# CDCA7 facilitates MET1-mediated CG DNA methylation maintenance in centromeric heterochromatin via linker histone H1

**DOI:** 10.1073/pnas.2526408122

**Published:** 2025-12-10

**Authors:** Shuya Wang, Tong Li, Matthew Naish, Russell Chuang, Evan K. Lin, Christian Fonkalsrud, Yan He, Suhua Feng, Ian R. Henderson, Steven E. Jacobsen

**Affiliations:** ^a^Molecular Biology Institute, University of California, Los Angeles, CA 90095; ^b^Department of Molecular, Cell and Developmental Biology, University of California, Los Angeles, CA 90095; ^c^Department of Plant Sciences, University of Cambridge, Cambridge CB2 3EA, United Kingdom; ^d^Eli and Edythe Broad Center of Regenerative Medicine and Stem Cell Research, University of California at Los Angeles, Los Angeles, CA 90095; ^e^HHMI, University of California, Los Angeles, CA 90095

**Keywords:** epigenetics, DNA methylation, centromere, genome stability, linker histone H1

## Abstract

DNA methylation is a conserved epigenetic modification essential for maintaining genome stability. However, how methyltransferases maintain CG methylation within compact chromatin, including centromeres, remains unclear. In humans, CDCA7 is necessary for the inheritance of DNA methylation at juxta-centromeres. Mutations that impair its ability to bind chromatin result in Immunodeficiency, Centromeric Instability, and Facial Anomalies (ICF) syndrome, characterized by centromeric instability. To investigate whether CDCA7 function is conserved, we identified two *Arabidopsis thaliana* orthologs, *CDCA7α* and *CDCA7β*. The loss of both copies results in CG hypomethylation at pericentromeric regions and centromeric satellite repeat arrays. Machine learning analysis suggested that heterochromatic nucleosomes, with enrichment of H1, H2A.W, and H3K9me2, depend heavily on CDCA7 proteins for CG methylation maintenance of the associated DNA. Loss of H1 restores heterochromatic DNA methylation in *cdca7α cdca7β* mutants, indicating that *CDCA7α* and *CDCA7β* mainly remodel H1-containing nucleosomes for methyltransferases to access DNA. Notably, in *h1.1 h1.2* mutants, CG methylation shows a significant increase in centromeres, which reveals a new inhibitory role of H1 in DNA methylation maintenance within satellite repeat arrays. Centromeric DNA hypermethylation is lost in *h1.1 h1.2 cdca7α cdca7β* quadruple mutants, demonstrating that *CDCA7α* and *CDCA7β* can act independently of H1 to enhance MET1 activity at nucleosomes. Overall, these findings establish *CDCA7α* and *CDCA7β* as conserved regulators of DNA methylation within heterochromatin and centromeric satellite repeat arrays.

## Significance

A key question in epigenetic memory is how DNA methylation is maintained and inherited within tightly packed heterochromatin. This process is crucial for genome stability, and its disruption can cause ICF syndrome, a disorder associated with mutations in CDCA7. Our study explains the conserved mechanism by which CDCA7 functions in *Arabidopsis*. We demonstrate that CDCA7 orthologs are vital for counteracting the inhibitory effect of linker histone H1 on the DNA methyltransferase MET1. More importantly, we also identify a second, H1-independent function in which CDCA7s directly boost MET1 activity at centromeric satellite repeats. These discoveries reveal a sophisticated, bimodal system that guarantees the faithful inheritance of CG methylation and deepen our understanding of the interplay between chromatin accessibility and epigenetic memory.

DNA methylation plays an essential role in silencing genes and transposable elements (TEs) (1), which is indispensable for development and reproduction in mammals (1–3). In *Arabidopsis thaliana* (hereafter *Arabidopsis*), DNA methylation occurs in CG, CHG (H = A, T, or C), and CHH sequence contexts. VIM proteins recognize hemimethylated cytosines and recruit MET1 to maintain CG methylation, while non-CG methylation is propagated by CHROMOMETHYLASE2 (CMT2) and CHROMOMETHYLASE3 (CMT3) (4). The RNA-directed DNA methylation (RdDM) pathway de novo methylates TEs via methyltransferases DRM1 and DRM2 (5). DNA methylation maintenance faces a significant challenge in regions with a high density of nucleosomes, particularly in heterochromatin, which restricts access to un- or hemimethylated cytosine substrates for DNA methyltransferases (6–9). To overcome this barrier, *Arabidopsis* DDM1 has been proposed to remodel H1-containing nucleosomes, enabling DNA methyltransferases to access pericentromeric chromatin (6, 7, 10–12). However, the mechanism guiding DDM1 to its nucleosome targets remains unclear.

In mammals, HELLS (the homolog of DDM1) depends on CDCA7 for localization and activation (13). Evolutionary analyses have suggested that CDCA7 is an essential cofactor for HELLS/DDM1 across eukaryotes (14). CDCA7 contains an evolutionarily conserved zf-4CXXC_R1 domain that recognizes hemimethylated CpG in the DNA major groove (13–17). Simultaneously, CDCA7 can interact with HELLS and relieve its catalytic autoinhibition (13). Current research suggests that CDCA7 recruits HELLS to satellite DNA arrays via the zf-4CXXC_R1 domain and activates HELLS to remodel nucleosomes for replication-uncoupled DNA methylation maintenance via UHRF (an ortholog of VIM) and DNMT1 (an ortholog of MET1) (15). Mutations in the zf-4CXXC_R1 domain, or HELLS-interacting domain, of CDCA7 lead to DNA hypomethylation at juxta-centromeric satellite DNA, resulting in centromere instability and ICF syndrome (13, 18). Mammalian centromeres consist of megabase arrays of alpha-satellite repeats, which serve as the site for CENP-A histone loading and kinetochore formation (19–22). Although the *Arabidopsis* genome is smaller (~130 Mb) compared to the human genome (~3 Gb), it also uses comparably sized megabase arrays of a 178-base pair satellite repeat (*CEN178*) for its centromeres (23–25). Centromeric and pericentromeric regions are heavily methylated at CG sites in both mammals and *Arabidopsis*. However, they differ in that CENP-A-occupied repeats are CG hypomethylated in mammals, whereas *Arabidopsis* CENH3-occupied *CEN178* repeats are densely CG methylated (22, 23). Whether CDCA7 proteins operate through similar mechanisms to assist CG methylation maintenance in plant centromeres and other genomic regions remains unknown. Additionally, DDM1 can remodel nucleosomes independently, while HELLS requires CDCA7 binding to perform remodeling activities (11, 26). This indicates that *Arabidopsis* CDCA7 proteins may function differently from their mammalian counterparts during DNA methylation maintenance.

In this study, we show that two Class I CDCA7 proteins, *CDCA7α* and *CDCA7β,* work redundantly to maintain CG methylation in *Arabidopsis* centromeric satellite repeat arrays and pericentromeric regions. The CG hypomethylation seen in *cdca7α cdca7β* mutants is less severe than in the *ddm1-2* and *met1* mutants, suggesting that *CDCA7α* and *CDCA7β* partially contribute to DDM1-dependent methylation. Machine learning analysis identified nucleosome density, linker histone H1 enrichment, plant-specific heterochromatic H2A variant H2A.W abundance, and H3K9me2 levels as key chromatin features predicting methylation loss in the *cdca7α cdca7β* background, indicating that compact chromatin depends heavily on *CDCA7α* and *CDCA7β* for CG methylation maintenance. Supporting this, H1 depletion restores DNA methylation in *cdca7α cdca7β* mutants, showing that H1 is the main barrier to VIM and MET1 access when CDCA7 proteins are absent. Therefore, in the wild-type, *CDCA7α* and *CDCA7β* act on H1-containing nucleosomes to promote the access of VIM and MET1. To determine whether CDCA7 functions independently of H1, we examined the *h1.1 h1.2* mutant. We found significant centromeric CG hypermethylation, revealing an unexpected role for H1 in preventing CG methylation within the *Arabidopsis* centromere satellite repeat arrays. This DNA hypermethylation is abolished in *h1.1 h1.2 cdca7α cdca7β* quadruple mutants, indicating that *CDCA7α* and *CDCA7β* also promote CG methylation even in the absence of H1. These findings highlight the conserved role of CDCA7 orthologs and demonstrate that *Arabidopsis* CDCA7 supports MET1 activity across a range of nucleosome contexts, including centromere satellite repeat arrays. Our results also provide genetic evidence that histone H1 regulates DNA methylation levels in centromeric satellite repeat arrays.

## Results

### *CDCA7α* and *CDCA7β* Are Conserved CDCA7 Orthologs.

**Fig. 1.**
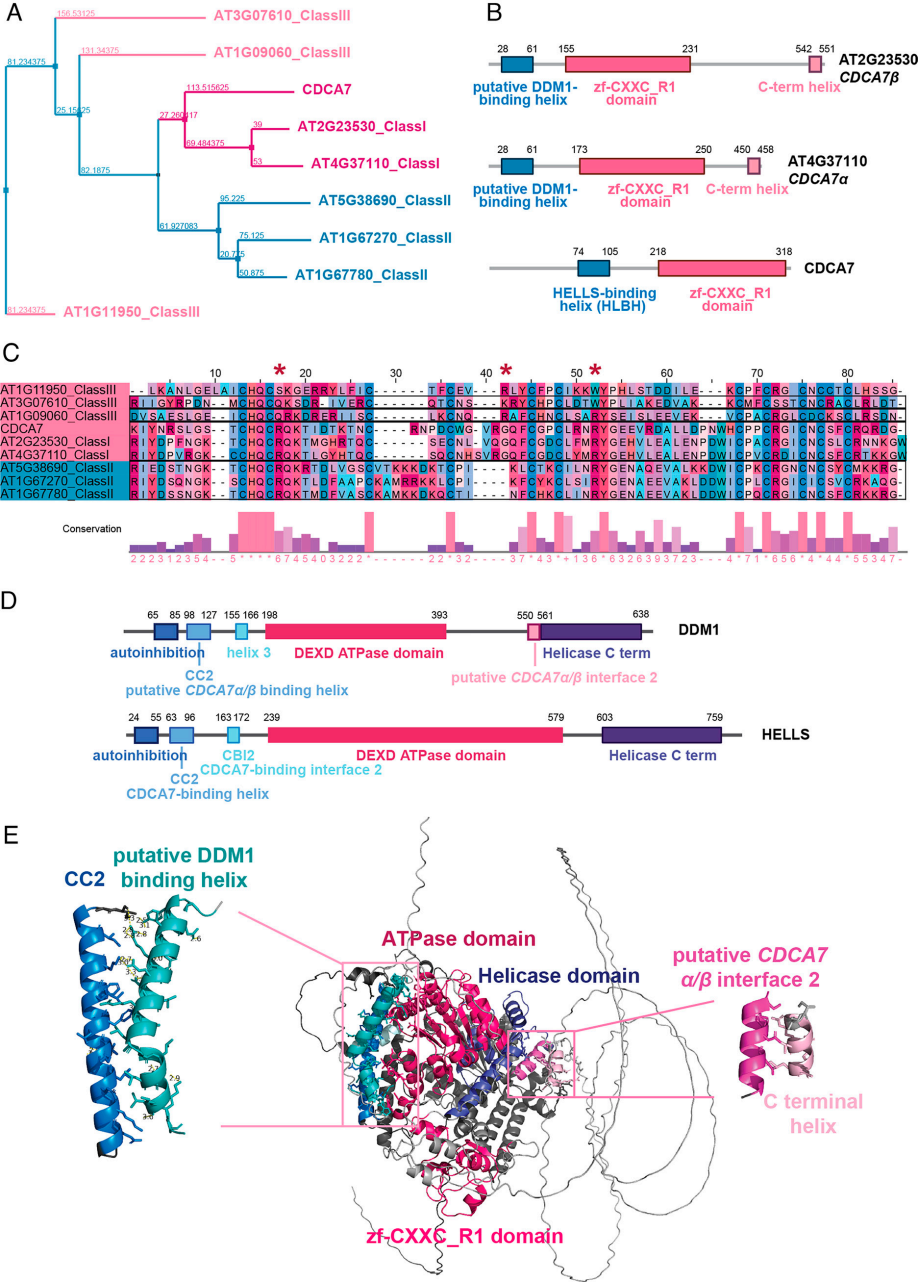
*CDCA7α* and *CDCA7β* are conserved CDCA7 orthologs (*A*) Phylogenetic tree based on the conservation of zf-CXXC_R1 domains of *homo sapiens* CDCA7 and its *Arabidopsis thaliana* homologs. (*B*) Functional domain annotations of *homo sapiens* CDCA7 and two *Arabidopsis* Class I CDCA7s. (*C*) Sequence alignment calculated by Clustal Omega of the zf-CXXC_R1 domains of *homo sapiens* CDCA7 and its *Arabidopsis* homologs. The red asterisks indicate key residues (R274, G294, and R304) that were mutated in ICF syndrome. (*D*) Domain annotations of HELLS and DDM1. (*E*) *CDCA7β* and DDM1 interaction model predicted by AF3. Cyan represents the putative DDM1-binding helix of *CDCA7β*. Blue represents CC2 of DDM1. Light pink represents the C-terminal helix of *CDCA7β*. Pink represents putative CDCA7 interface 2 of DDM1. Dark pink represents the ATPase domain of DDM1. Purple indicates the Helicase C term domain of DDM1. Bright pink represents the zf-CXXC_R1 domain of *CDCA7β*.

Mutations in three critical amino acids (R274, G294, and R304) of the CDCA7 zf-CXXC_R1 domain reduce its binding affinity to hemimethylated CpG sites, leading to ICF syndrome (15). Among the three classes of *Arabidopsis* CDCA7 proteins (Fig. 1*A*), only Class I CDCA7s (*CDCA7α* and *CDCA7β*) retain these residues and the cysteines required for zf-CXXC_R1 domain folding (Fig. 1 *B* and *C*) (14). Consistent with this conservation, *CDCA7α* (AT4G37110) and *CDCA7β* (AT2G23530) preferentially bind hemimethylated DNA over fully methylated or unmethylated DNA in vitro, suggesting a similar selectivity in vivo (15)

Class I CDCA7s also share a similar protein structure with mammalian CDCA7, including an N-terminal helix analogous to the HELLS-binding helix (HLBH) (Fig. 1*B* and *SI Appendix*, Fig. S1*A*). Both HELLS and *Arabidopsis* DDM1 have an N-terminal coiled-coil domain (CC2), which is known in HELLS to be critical for interaction with CDCA7 (Fig. 1*D*) (11, 15). AlphaFold 3 (AF3) predicts that the HLBH of *CDCA7α* and *CDCA7β* directly interacts with DDM1’s CC2 through multiple types of interactions (Fig. 1*E* and *SI Appendix*, Fig. S1 *B*–*D* and Tables S1 and S2) (27). Consistent with this, DDM1 lacking the CC2-containing N-terminal domain cannot complement *ddm1* mutants (12), indicating that interaction with *CDCA7α*/*β* is essential for DDM1 function. In addition to the HLBH–CC2 interaction, HELLS also relies on the CDCA7-binding interface 2 (CBI2) to strengthen its association with CDCA7. By contrast, DDM1 is predicted to use its helicase domain to interact with the C-terminal helix of *CDCA7α*/*β* (Fig. 1*E* and *SI Appendix*, Fig. S1*D*). This interaction likely compensates for the absence of CBI2, suggesting different recruitment strategies of CDCA7 proteins in plants and mammals. Overall, AF3’s structural predictions suggest that *CDCA7α* and *CDCA7β* retain key features of human CDCA7 and imply their functional conservation.

### *CDCA7α* and *CDCA7β* Are Required for the Maintenance of Heterochromatic DNA Methylation.

**Fig. 2.**
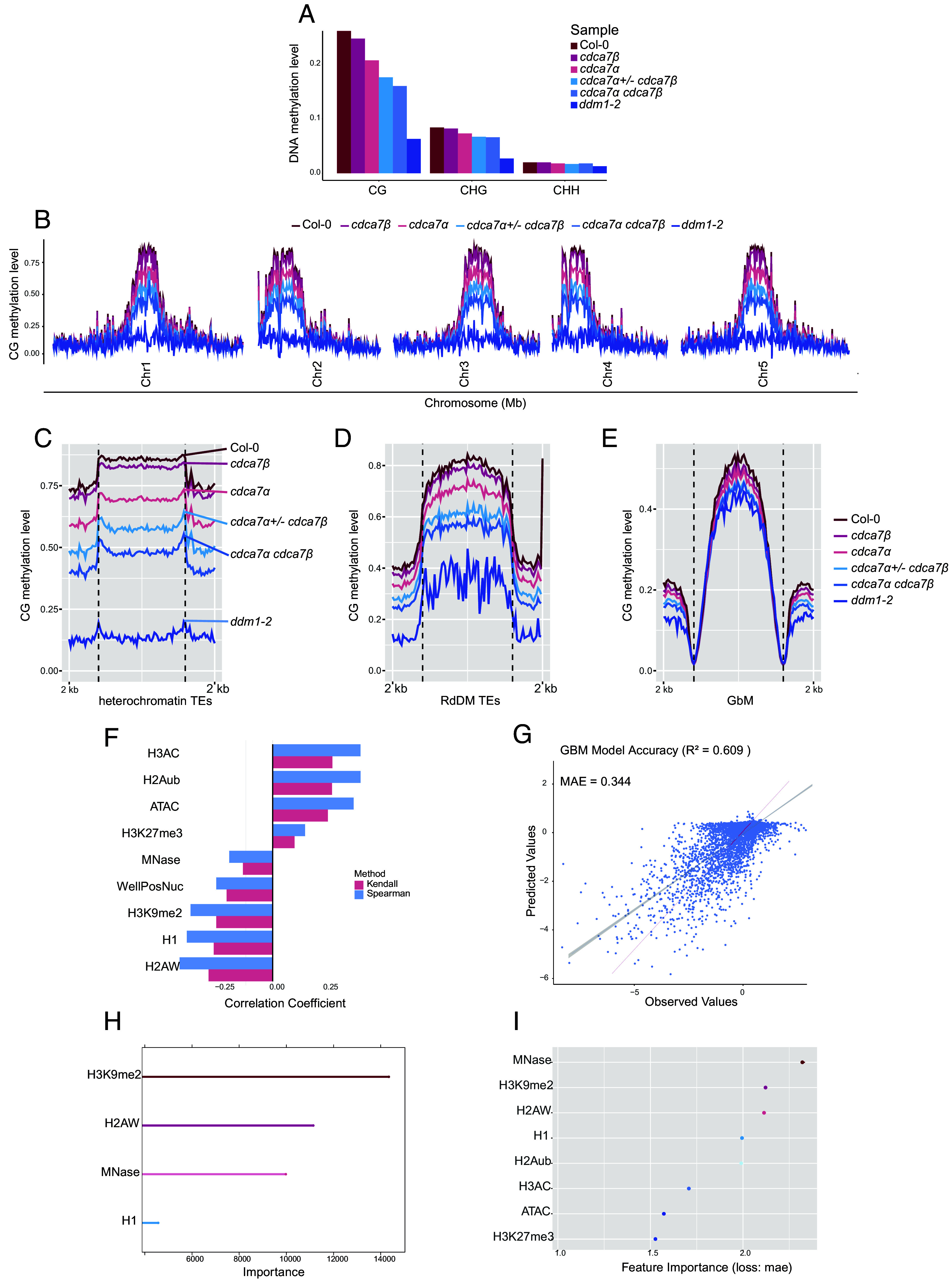
*CDCA7α* and *CDCA7β* maintain heterochromatic DNA methylation (*A*) Global DNA methylation summary of Col-0, *cdca7α, cdca7β, cdca7α*^+/−^ *cdca7β, cdca7α cdca7β*, and *ddm1-2*. (*B*) Genome-wide CG methylation landscape of Col-0*, cdca7α, cdca7β, cdca7α^+/−^ cdca7β, cdca7α cdca7β,* and *ddm1-2*. Metaplots showing the CG methylation level at (*C*) heterochromatin TEs, (*D*) RdDM targeted TEs, and (*E*) GbM across genotypes. (*F*) Spearman and Kendall correlation coefficients between epigenetic features and CG methylation changes in the *cdca7α cdca7β* mutant compared to wild type. (*G*) Prediction accuracy of generalized boosted regression model. (*H*) Rank of the importance of epigenetic features via the generalized boosted regression model. (*I*) The rank of the importance of epigenetic features, derived by shuffling each feature and measuring the reduction in performance.

Given the evolutionary conservation between *Arabidopsis CDCA7α and CDCA7β* homologs and mammalian CDCA7, we investigated their roles in DNA methylation maintenance. Using CRISPR-Cas9, we generated loss-of-function mutants for *cdca7α, cdca7β, cdca7α*^+/−^ *cdca7β,* and *cdca7α cdca7β* in the Col-0 background (*SI Appendix*, Fig. S2*A*) and conducted Whole-Genome Bisulfite Sequencing (WGBS) to assess genome-wide DNA methylation. Loss of *CDCA7β* alone results in a 2% reduction in overall CG methylation, primarily observed in heterochromatin (Fig. 2 *A*–*E*). In comparison, knocking out *CDCA7α* alone causes a 6% decrease in CG methylation at pericentromeric regions (Fig. 2 *A*−*E*), while non-CG methylation shows less than 1% difference (Fig. 2*A* and *SI Appendix*, Fig. S2 *B* and *C*). These findings suggest that *CDCA7α*, compared to *CDCA7β*, plays a more prominent role in supporting VIM and MET1-mediated CG methylation.

Since *CDCA7α* and *CDCA7β* may be genetically redundant, we knocked out one *CDCA7α* allele in the *CDCA7β* mutant background. Lacking one *CDCA7α* copy enhanced the *cdc7β* CG hypomethylation phenotype (Fig. 2 *A*–*E*), while complete knockout of both *CDCA7α* and *CDCA7β* caused severe CG methylation loss at heterochromatic TEs (40% loss), compared to RdDM-targeted TEs (25% loss), and gene-body methylated (GbM) genes (10% loss). This reveals a genetic redundancy and a dosage-dependent role for *CDCA7α*. In contrast, CHG and CHH methylation decreased only slightly at heterochromatin (less than 10% loss) (*SI Appendix*, Fig. S2 *B* and *C*). Notably, *cdca7α cdca7β* mutants retained approximately 50% of wild-type pericentromeric CG methylation, which is much higher than the 10% in *ddm1-2* mutants and the 2% (near-complete loss) in *met1* mutants (Fig. 2 *A*–*E*) (28). Therefore, unlike CDCA7 in mammals, *CDCA7α* and *CDCA7β* are only partially required for DDM1 function in vivo. DDM1 may either remodel nucleosomes independently of *CDCA7α*/*β* or depend on additional methylation readers for recruitment and activity.

To further analyze the genome-wide patterns of DNA CG hypomethylation in *cdca7α cdca7β* mutants, we mapped hypomethylated differentially methylated regions (hypoDMRs). These regions were mainly enriched in pericentromeric chromatin, which is characterized by high nucleosome density, H2A.W, H3K9me2 marks, and linker histone H1 (*SI Appendix*, Fig. S2 *D* and *E*). Supporting this, heterochromatic nucleosomes show about 27% CG methylation loss, while genic nucleosomes only exhibit a 3% reduction in *cdca7α cdca7β* (*SI Appendix*, Fig. S2 *F* and *G*). This suggests that heterochromatic nucleosomes, in part, depend on *CDCA7α* and *CDCA7β* for CG methylation maintenance.

To identify epigenetic features that predict CG methylation loss, we combined correlation analysis, machine learning, and feature importance ranking. Spearman and Kendall correlation coefficients showed strong links between *cdca7α cdca7β* CG hypomethylation and the heterochromatic features H2A.W, H1, and H3K9me2 (Fig. 2*F* and *SI Appendix*, Fig. S2*H*). Conversely, transcription-activating marks like H3AC and H2Aub, along with ATAC-seq signals (indicating open chromatin) in wild-type, were associated with increased CG methylation in *cdca7α cdca7β* mutants (*SI Appendix*, Fig. S2 *E* and *H*). This increase in CG DNA methylation occurs at open chromatin regions, consistent with the redistribution of VIMs and MET1 to more accessible euchromatin when heterochromatin access is restricted due to the loss of *CDCA7α* and *CDCA7β*.

Using a generalized boosted regression model to predict CG methylation loss in *cdca7α cdca7β*, we achieved an *R^2^* value of 0.609 and a mean absolute error of ~0.3 (Fig. 2*G*). Among all features, H3K9me2, H2A.W, and nucleosome density (MNase-seq signal) emerged as the top predictors of CG hypomethylation in *cdca7α cdca7β* (Fig. 2*H*). To account for multicollinearity among features, we employed a permutation-based approach, shuffling individual features and quantifying their impact on model performance. Nucleosome density (MNase-seq) was the most critical predictor, followed by H3K9me2, H2A.W, and H1 enrichment (Fig. 2*I*). These findings suggest that the CG maintenance machinery requires *CDCA7α* and *CDCA7β* to enable access to heterochromatic nucleosomes marked by H3K9me2, H2A.W, and H1 for CG methylation maintenance. The interplay of these features underscores the chromatin context dependency of *CDCA7α*/*β* mediated CG methylation.

### *CDCA7α* and *CDCA7β* Facilitate Methyltransferase Activity At H1-Containing Nucleosomes.

**Fig. 3.**
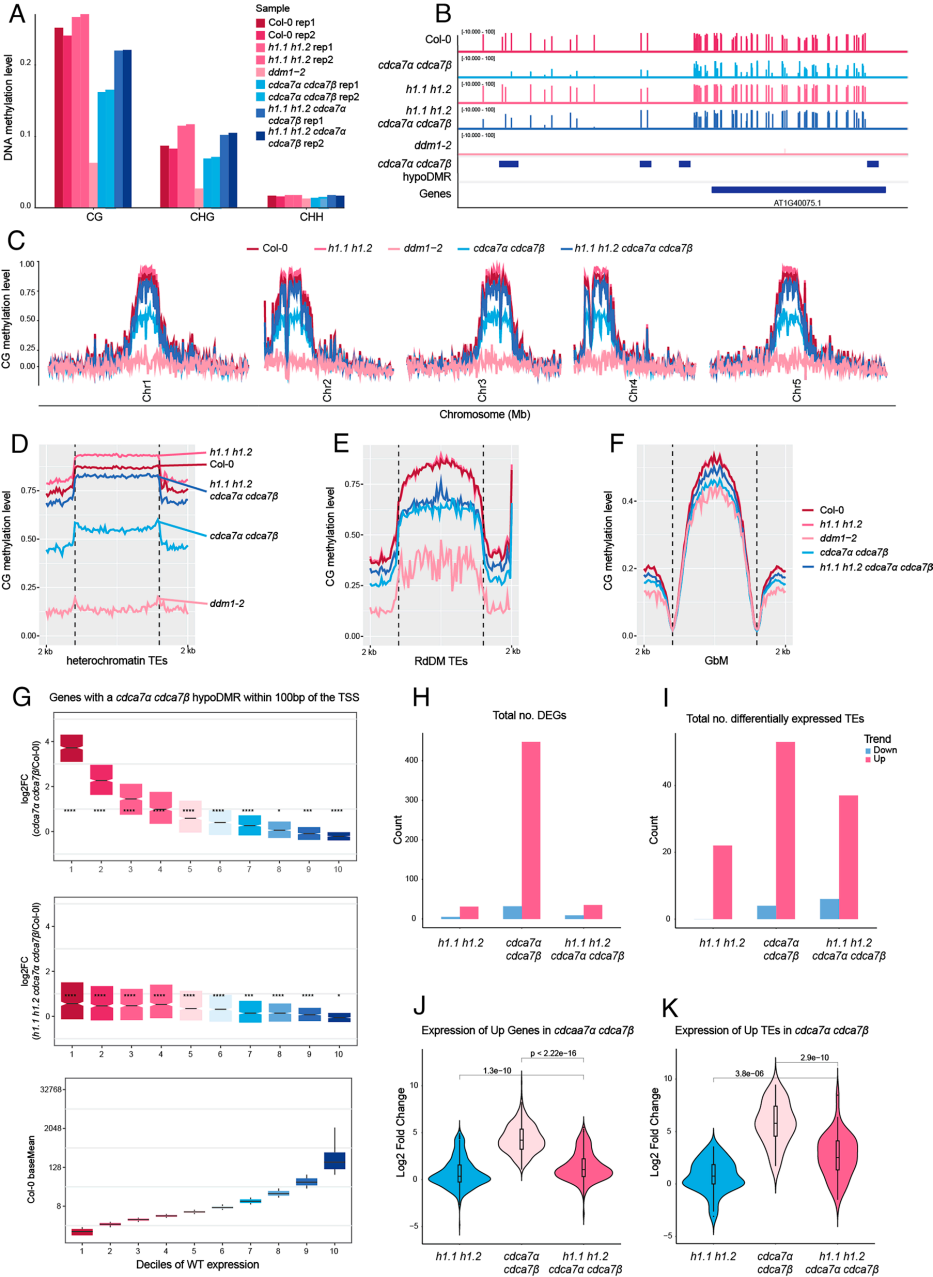
*CDCA7α* and *CDCA7β* facilitate methyltransferases to access H1-containing nucleosomes (*A*) Global DNA methylation summary of Col-0, *h1.1 h1.2, cdca7α cdca7β, h1.1 h1.2 cdca7α cdca7β,* and *ddm1-2*. (*B*) Genome browser example showing CG methylation levels at the representative locus in Col-0, *cdca7α cdca7β, h1.1 h1.2, h1.1 h1.2 cdca7α cdca7β,* and *ddm1-2*. (*C*) Genome-wide CG methylation landscape of Col-0, *cdca7α cdca7β, h1.1 h1.2, h1.1 h1.2 cdca7α cdca7β*, and *ddm1-2*. Metaplots showing the CG methylation level at (*D*) heterochromatin TEs, (*E*) RdDM targeted TEs, and (*F*) GbM across genotypes. (*G*) Normalized expression of genes proximal to the *cdca7α cdca7β* hypoDMR. The *Bottom* bar plot shows the ten deciles clustered by wild-type expression level. Total number of differentially expressed (*H*) genes, and (*I*) TEs in *h1.1 h1.2, cdca7α cdca7β*, and *h1.1 h1.2 cdca7α cdca7β*. Log2 fold change of expression of upregulated (*J*) genes, and (*K*) TEs in *h1.1 h1.2, cdca7α cdca7β*, and *h1.1 h1.2 cdca7α cdca7β*.

DDM1 is essential for CG DNA methylation activity in H1-containing chromatin (6, 7). To determine whether *CDCA7α*/*β* facilitates DNA methyltransferase activity at H1-containing nucleosomes, we generated *h1.1 h1.2 cdca7α cdca7β* quadruple mutants (*SI Appendix*, Fig. S3*A*) and compared CG methylation levels to those in *cdca7α cdca7β* mutants. Genome-wide CG hypomethylation in the *cdca7α cdca7β* mutants was largely restored in the quadruple mutants, with DNA methylation levels nearly returning to wild-type at heterochromatic TEs, RdDM-targeted TEs, and GbM genes (Fig. 3 *A*–*F*). These findings support that *CDCA7 α/β* and DDM1 operate in the same pathway for DNA methylation maintenance, playing a role in counteracting the repression of methyltransferase activity caused by histone H1 enrichment.

We found that a subset of hypoDMRs, located at heterochromatin-euchromatin boundaries, remained DNA hypomethylated in *h1.1 h1.2 cdca7α cdca7β* mutants (*SI Appendix*, Fig. S3*B*). These regions showed lower nucleosome density, reduced chromatin accessibility, and depletion of repressive histone marks (*SI Appendix*, Fig. S3 *C*–*F*), indicating that *CDCA7α* and *CDCA7β* target inaccessible heterochromatin edges. We also found that MORC proteins, ATP-dependent enzymes that compact and silence chromatin (29), preferentially target regions that remain hypomethylated in *h1.1 h1.2 cdca7α cdca7β* mutants (*SI Appendix*, Fig. S3*G*). Therefore, outside of H1-enriched nucleosomes, *CDCA7α* and *CDCA7β* may assist VIM-MET1 to overcome MORC-mediated chromatin compaction.

CG hypomethylation at *cdca7α cdca7β* hypoDMRs triggered mRNA upregulation of proximal genes (within 100 bp), especially those with low baseline expression in wild type (Fig. 3*G*). Notably, H1 depletion restored the expression of most *cdca7α cdca7β* upregulated genes to near-wild type levels (Fig. 3*G*). Beyond the genes proximal to *cdca7α cdca7β* hypoDMRs, genome-wide, *cdca7α cdca7β* mutants activated approximately 450 genes, with most (85%) becoming transcriptionally silent in *h1.1 h1.2 cdca7α cdca7β* (Fig. 3*H*). Additionally, about 55 TEs became significantly activated in *cdca7α cdca7β* (Fig. 3*I*). A subset of the *cdca7α cdca7β* activated TEs became downregulated by H1 loss, while TEs close to the remaining *h1.1 h1.2 cdca7α cdca7β* hypoDMRs remained upregulated compared to wild type (Fig. 3*I* and *SI Appendix*, Fig. S3*H*). When examining the extent of gene and TE transcript upregulation, the loss of H1 significantly reduced the degree of upregulation at activated genes, and also partially at activated TEs (Fig. 3 *J* and *K*), consistent with the degree of DNA methylation rescue. These findings demonstrate that *CDCA7α* and *CDCA7β* mainly operate in H1-enriched heterochromatin to preserve DNA methylation and enforce transcriptional silencing. Their ability to act at heterochromatin boundaries, independent of H1, also highlights a context-specific role.

### *CDCA7α* and *CDCA7β* Promote DNA Methylation At Centromeres.

**Fig. 4.**
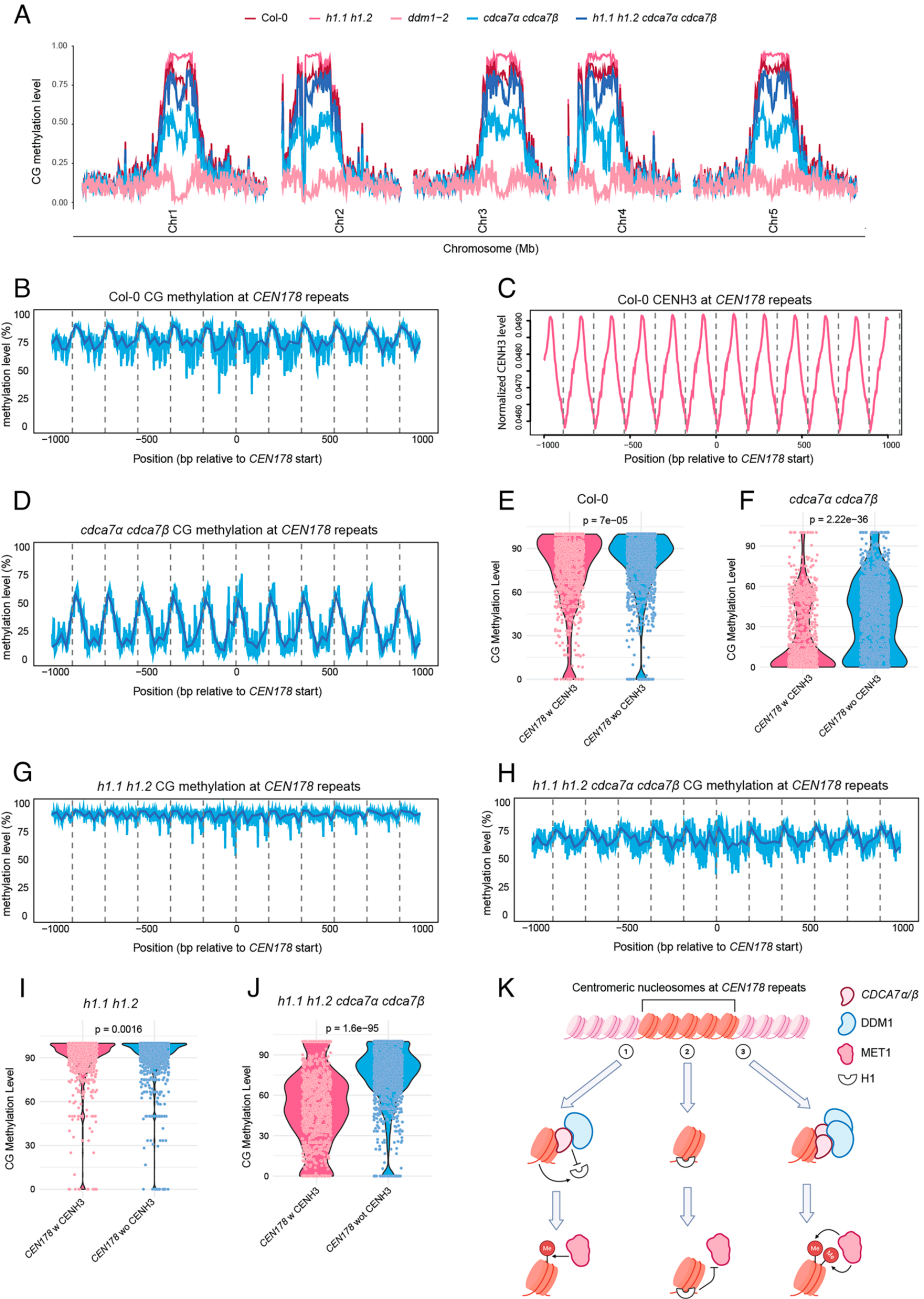
*CDCA7α* and *CDCA7β* promote CG methylation establishment at centromeres. (*A*) Global CG methylation summary of Col-0, *cdca7α cdca7β, h1.1 h1.2, h1.1 h1.2 cdca7α cdca7β,* and *ddm1-2*, using Col-Cen-v1.2 as the reference genome. (*B*) Metaplot showing the CG methylation level at *CEN178* satellite repeats in Col-0. (*C*) Metaplot showing the normalized wild-type CENH3 enrichment at the *CEN178* satellite repeats. (*D*) Metaplot showing the CG methylation level at *CEN178* satellite repeats in the *cdca7α cdca7β* mutant. Violin plots showing the CG methylation level at *CEN178* satellite repeats with and without CENH3 enrichment in (*E*). Col-0, and (*F*) *cdca7α cdca7β.* Metaplots showing the CG methylation level at *CEN178* satellite repeats in (*G*) *h1.1 h1.2* and (*H*) *h1.1 h1.2 cdca7α cdca7β.* Violin plots showing the CG methylation level at *CEN178* satellite repeats with and without CENH3 enrichment in (*I*) *h1.1 h1.2*, and (*J*) *h1.1 h1.2 cdca7α cdca7β* mutants. (*K*) Schematic of the proposed mechanism of *CDCA7α*/*β* at centromeric regions. (1) represents the wild-type condition when *CDCA7α*/*β* recruits DDM1 to remodel H1-containing nucleosomes for VIM- and MET1-mediated CG methylation maintenance. (2) shows that MET1 access to nucleosomes is limited by H1 in the *cdca7α cdca7β* mutant. (3) demonstrates that when H1 is depleted, *CDCA7α*/*β* promotes CG methylation by reducing the nucleosome barrier for MET1. Made via BioRender.

Since ICF syndrome-associated CDCA7 mutations cause DNA hypomethylation at human centromere alpha-satellite repeats (13, 18), we hypothesized that *Arabidopsis CDCA7α* and *CDCA7β* might similarly regulate repetitive regions, including centromeric *CEN178* satellite repeats. To test this, we analyzed DNA methylation at centromeric regions using the Col-CEN-v1.2 genome assembly (23), which fully resolves centromeric sequences. Loss of *CDCA7α* and *CDCA7β* resulted in approximately a 50% reduction in CG methylation at satellite repeats, while non-CG methylation was only modestly affected (15%) (Fig. 4*A* and *SI Appendix*, Fig. S4 *A* and *B*). Within the *CEN178* repeat arrays, not only was CG methylation substantially reduced, but its distribution also shifted toward linker DNA regions (Fig. 4 *B*–*D*). This pattern suggests that *CDCA7α* and *CDCA7β* are required for proper DNA methylation distribution at CENH3-containing nucleosomes. Supporting this, the decrease in CG methylation was more pronounced at *CEN178* repeats with higher CENH3 enrichment (Fig. 4 *E* and *F*).

We next assessed whether H1 depletion in the *cdca7α cdca7β* mutant background could similarly restore DNA methylation loss, as we previously observed in the chromosome arms and pericentromeres. Compared to *cdca7α cdca7β* mutants, *h1.1 h1.2 cdca7α cdca7β* quadruple mutants gained CG methylation within the *CEN178* satellite arrays, but only partially (60% versus 80% in wild-type), indicating that *CDCA7α* and *CDCA7β* function beyond H1 in the centromeres (Fig. 4*A*). This aligns with the incomplete rescue of DNA methylation at heterochromatin boundaries seen in *h1.1 h1.2 cdca7α cdca7β* mutants (*SI Appendix*, Fig. S3*B*).

To directly examine the role of *CDCA7α* and *CDCA7β* in centromeric CG methylation without H1, we compared *h1.1 h1.2* and *h1.1 h1.2 cdca7α cdca7β* mutants. Notably, CG methylation, and to a lesser extent, non-CG methylation, increased significantly with H1 loss (Fig. 4*A* and *SI Appendix*, Fig. S4 *A* and *B*). This aligns with DNA hypermethylation observed at heterochromatin in the *h1.1 h1.2* mutant. At *CEN178* satellite arrays, CG methylation approaches nearly 100% in *h1.1 h1.2*, indicating that H1 acts as a barrier to DNA methylation in *Arabidopsis* centromeres (Fig. 4 *A*–*C*, and *G*). In the *h1.1 h1.2* mutants, CG methylation was primarily gained in the centers of *CEN178* repeats, which are typically enriched for CENH3 (Fig. 4 *B*, *C*, and *G*). This CG hypermethylation in the middle of the *CEN178* repeats was lost in the *h1.1 h1.2 cdca7α cdca7β* mutants (Fig. 4 *C*, *G*, and *H*). Consistently, the CG methylation decrease in the quadruple mutant was more pronounced within CENH3-enriched *CEN178* repeats, further emphasizing the role of *CDCA7α* and *CDCA7β* in maintaining DNA methylation at centromeric nucleosomes (Fig. 4 *I* and *J*). However, the increase in CHG and CHH DNA methylation in *h1.1 h1.2* was unaffected by the loss of *CDCA7α* and *CDCA7β* (*SI Appendix*, Fig. S4 *A*–*F*), indicating that they specifically promote VIM and MET-mediated CG methylation.

We propose that the depletion of linker histone H1 broadly decompacts heterochromatin, allowing increased access for *CDCA7α* and *CDCA7β* to the centromeres, thereby promoting CG hypermethylation by VIM and MET1 through a replication-uncoupled mechanism (Fig. 4*K*), to compensate for imperfect replication-coupled maintenance (15, 30). However, the mechanism by which *CDCA7α* and *CDCA7β* are recruited to centromeres remains unclear. One possibility is that the conserved zf-CXXC_R1 domain in *CDCA7α* and *CDCA7β* recognizes non-B-DNA structures in centromeric satellites, as shown in vitro (17). Alternatively, *CDCA7α* and *CDCA7β* might directly recognize *CEN178* repeats or associated centromeric chromatin marks, diverging from the binding preferences seen in mammalian CDCA7.

## Discussion

This work indicates that *CDCA7α* and *CDCA7β*, the *Arabidopsis* counterparts of mammalian CDCA7, interact with DDM1 to help maintain CG methylation, which is consistent with a recent publication on *CDCA7α* and *CDCA7β* (31). Differing from mammalian studies, we suggest that *CDCA7α* and *CDCA7β* facilitate VIM and MET1 access to tightly packed heterochromatin by remodeling H1-containing nucleosomes at pericentromeric regions. This idea is supported by the rescue of pericentromeric DNA hypomethylation in *cdca7α cdca7β* mutants when H1 is lost in *h1.1 h1.2 cdca7α cdca7β* quadruple mutants. We also found a role for *CDCA7α* and *CDCA7β* in promoting centromeric CG methylation in the absence of H1. We suggest that *CDCA7α* and *CDCA7β* remodel CENH3-containing nucleosomes and provide better access to methyltransferases, including but not limited to VIM and MET1 (Fig. 4*K*).

Importantly, our study uncovers a role for H1 in preventing DNA methylation at centromeric chromatin, a function not previously reported. Since the loss of H1 results in nearly saturated CG methylation at satellite repeats, primarily due to *CDCA7α* and *CDCA7β* activity, we suggest that H1-bound hemimethylated nucleosomes are common features of *Arabidopsis* centromeres. H1 depletion then allows DNA methylation maintenance through the CDCA7 pathway. This finding provides insight into the mechanism behind the low DNA methylation levels at mammalian CENP-A-occupied repeats, with a high abundance of linker histone H1 likely restricting centromeric DNA methylation. *CDCA7α* and *CDCA7β* may directly recognize centromeric DNA motifs to facilitate methylation establishment and maintenance in these regions. The conserved zf-CXXC_R1 domain of *CDCA7α* and *CDCA7β* can bind both canonical and non-B-DNA structures (17), but its exact targeting preferences remain unknown. Future studies combining in vitro binding assays and in vivo mutagenesis will clarify whether centromeric repeats or structural features determine *CDCA7α*/*β* localization.

While *cdca7α cdca7β* mutants show DNA hypomethylation, the extent of reduction is less than in *ddm1-2* and *met1* mutants. This difference in phenotypic severity suggests two possibilities: 1) *Arabidopsis* Class II/III CDCA7 homologs might partially compensate for *CDCA7α* and *CDCA7β* in recruiting DDM1, or 2) DDM1 can still partially localize to heterochromatin without CDCA7 proteins. Further research into DDM1 recruitment mechanisms is needed to clarify this. These findings underscore the conserved function of CDCA7 proteins in linking chromatin remodelers and methyltransferases across different species, while also revealing plant-specific changes in centromere regulation. By clarifying how *CDCA7α*/*β* control H1-dependent and H1-independent methylation, this work enhances our understanding of chromatin accessibility and DNA methylation dynamics.

## Materials and Methods

### Phylogenetic Analysis.

Highly conserved zf-CXXC_R1 domain sequences of *Arabidopsis* Class I (*CDCA7α* and *CDCA7β*), Class II (AT1G67270, AT1G67780, and AT5G38690), Class III (AT1G09060, AT1G11950, and AT3G07610) CDCA7, and human CDCA7 were used for phylogenetic analysis. The sequences are displayed in Fig. 1*C*. To compare the proteins, alignments were carried out with Clustal Omega. The evolutionary relationships were visualized by constructing a phylogenetic tree using Jalview.

### AlphaFold Prediction.

Full-length proteins of *CDCA7α/β* and DDM1 are taken for AlphaFold3 prediction (27). For each prediction, the best-performing model was selected for further structural examination. A cutoff distance of 5 Å was applied. The protein structures were visualized using Pymol.

### Plant Materials and Growth Conditions.

Plant growth conditions adhered to the previous publication (32). Briefly, plants utilized in this paper were *Arabidopsis thaliana* Col-0 ecotype and were grown under long-day conditions (16 h light and 8 h dark). Seedlings of the Col-0, *cdca7α, cdca7β*, *cdca7α^+/−^ cdca7β*, *cdca7α cdca7β*, *h1.1 h1.2*, *h1.1 h1.2 cdca7α cdca7β*, and *ddm1-2* were harvested after 14 d of incubation under long-day conditions. This study utilizes the T-DNA insertion lines, which are listed here: *h1.1* (SALK_128430C) and *h1.2* (GABI_406H11_012502). *ddm1-2* contains a splice donor site mutation. CRISPR mutants were produced using the pBEE401E CRISPR system (33). *CDCA7α* CRISPR mutant was generated using two combinations of guides: GGGGTTTCTTTGATTAGTTC and TTGGGAATACAGAAAGAAGC or CAAAGGTCTCTCTTTACGAA and ATCCCATCAGTGTAGATAAC. *CDCA7β* CRISPR mutant was generated using two combinations of guides: TTCGCTCTCGTTCTCACCAC and AAGGCCAGAGATTTACACTG or GAGTTTCCTCCTCCGACTGT and GGTTCCTCTGCGTAGGAAAC.

### WGBS.

WGBS was performed as described in the previous publication (32). In brief, 14-day-old seedlings were harvested from Col-0, *cdca7α, cdca7β*, *cdca7α^+/−^ cdca7β*, *cdca7α cdca7β*, *h1.1 h1.2*, *h1.1 h1.2 cdca7α cdca7β*, and *ddm1-2.* Samples were immediately frozen in liquid nitrogen. DNA extraction was performed using the DNeasy Plant Mini kit (Qiagen). A total of 100 ng of DNA was sheared to approximately 200 bp with the Covaris S2 (Covaris). Libraries were then prepared using the Ovation Ultralow Methyl-seq kit (NuGEN), followed by bisulfite conversion with the Epitect Bisulfite Conversion kit (QIAGEN). The libraries were finally sequenced on Illumina NovaSeq X Plus instruments.

### Correlation Analysis.

Epigenetic data were downloaded from published datasets. H1, H3K9me2, MNase-seq, H3AC, H2Aub, H3K27me3, H2A.W, and ATAC-seq were normalized using the preprocess function from the caret library. Then, correlation coefficients were calculated between variables using the Spearman and Kendall algorithms, provided by the corrplot package. Visualizations of the correlation matrix were performed using the ggplot2 package.

### Machine Learning.

Epigenetic features with high correlation (>0.78) were removed before data normalization. Then, 75% of data points were used for training, and the remaining 25% were used for testing. The Gradient Boosted Regression (GBM) model was selected based on its highest performance. A 10-fold cross-validation was repeated 10 times to examine model performance. Tuning parameters were applied as follows: interaction depth (3, 6, 9), number of trees (200, 250, 300). Finally, R-squared and MAE values were calculated, and the importance of epigenetic features was ranked.

### Feature Importance Ranking.

Feature importance measure was alternatively performed using the iml package. Each feature was shuffled, and the decrease in the GBM model performance was calculated. The loss in performance was measured with MAE.

### RNA-seq.

RNA-seq was performed according to the protocols described in a previous publication (32). Briefly, three biological replicates were prepared for each genotype. Each replicate consisted of a single 2-week-old seedling collected and frozen in liquid nitrogen. The samples were ground into powder, and RNA was extracted using the Direct-zol RNA MiniPrep kit (Zymo Research). For RNA-seq library construction, 500 ng of total RNA from the seedlings was used with the TruSeq Stranded mRNA kit (Illumina). The resulting library was sequenced on an Illumina NovaSeq X Plus instruments.

### WGBS Analysis.

WGBS analysis was done according to the pipeline described in previous publication (32). Reads were filtered and Illumina adaptors removed using Trim Galore (v 0.6.7, Babraham Institute). Reads with three or more consecutive methylated CHH sites were considered nonconverted and excluded from analysis. Bismark (v 0.19.1, Babraham Institute) (34) mapped the reads to the Arabidopsis reference genome (TAIR10) and Col-Cen-v1.2 assembly (23). ViewBS (v 0.1.11) generated the plots (35). To compute DNA methylation levels across 1 kb upstream and downstream of CEN178 centers, deepTools (v 3.0.2) (36) was used with the computeMatrix reference-point option. The resulting matrix was processed with custom Perl and R scripts to produce the final methylation profile plots.

### RNA-seq Analysis.

RNA-seq analysis was conducted following the pipeline outlined in a previous publication. (32). Reads were filtered, and Illumina adaptors were trimmed using Trim Galore (v 0.6.7, Babraham Institute). Left reads were aligned to the Arabidopsis reference genome (TAIR10) with STAR (v 2.7.11a) (37). Only reads that mapped uniquely with less than 5% mismatches were retained. For visualization, bigwig files were created using deeptools (v 3.0.2) (36) bamCoverage with --normalizeUsing RPGC and --binSize 10. Read counts for genes and TEs were obtained with HTSeq (v 0.13.5). Differential analysis was performed with DESeq2 (v 1.42.0), using a cutoff of padj < 0.05 and |log2FC| ≥ 1. Finally, all associated plots were generated using ggplot2 (v 3.4.4).

## Supplementary Material

Appendix 01 (PDF)

## Data Availability

The high-throughput sequencing data from this study are stored in the Gene Expression Omnibus (GEO) database. RNA-seq data (38) (accession no: GSE308805) can be accessed via this link: https://www.ncbi.nlm.nih.gov/geo/query/acc.cgi?acc=GSE308805. WGBS data (39) (accession no: GSE308807) is available at: https://www.ncbi.nlm.nih.gov/geo/query/acc.cgi?acc=GSE308807.
