# Supplementary material for: CDCA7 facilitates MET1-mediated CG DNA methylation maintenance in centromeric heterochromatin via linker histone H1
Source: Proc Natl Acad Sci U S A. 2025 Dec 10;122(50):e2526408122. doi: 10.1073/pnas.2526408122 (PMC12718391; doi:10.1073/pnas.2526408122)
Supplement: Supplementary file 1 — Appendix 01 (PDF) [file pnas.2526408122.sapp.pdf]

PNAS

Supporting Information for
CDCA7 facilitates MET1-mediated CG DNA methylation maintenance in centromeric heterochromatin via linker histone H1

Shuya Wang, Tong Li, Matthew Naish, Russell Chuang, Evan K. Lin, Christian Fonkalsrud, Yan He, Suhua Feng, Ian R. Henderson, and Steven E. Jacobsen

Correspondence: Ian R. Henderson and Steven E. Jacobsen
Email: irh25@cam.ac.uk and jacobsen@ucla.edu

This PDF file includes:

Figures S1 to S4
Tables S1 to S2

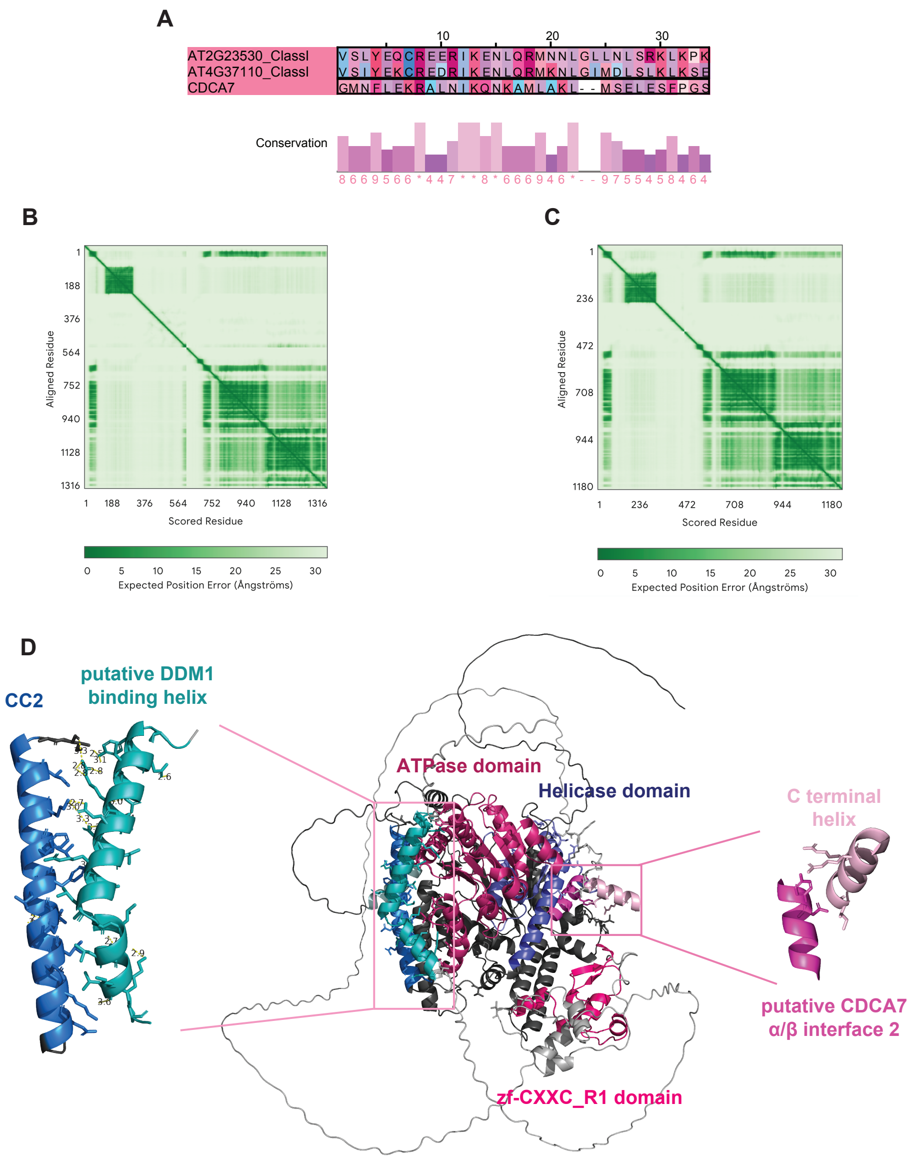

Fig. S1. *CDCA7α* and *CDCA7β* retain the HLBH domain for interacting with DDM1.

A. Sequence alignment calculated by Clustal Omega of the HLBH domains of *homo sapiens* CDCA7 and *Arabidopsis* Class I homologs. The red asterisk indicated the point mutations. Predicted Aligned Error (PAE) from AF3 structural modeling of **B.** *CDCA7β* or **C.** *CDCA7α* and DDM1 interactions. **D.** Close illustration of the AF3 predicted interface between *CDCA7α* and DDM1. Cyan represents the putative DDM1-binding helix of *CDCA7α*. Blue represents CC2 of DDM1. Light pink represents the C-terminal helix of *CDCA7α*. Pink represents putative CDCA7 interface 2 of DDM1. Dark pink represents the ATPase domain of DDM1. Purple indicates the Helicase C term domain of DDM1. Bright pink represents the zf-CXXC_R1 domain of *CDCA7α*.

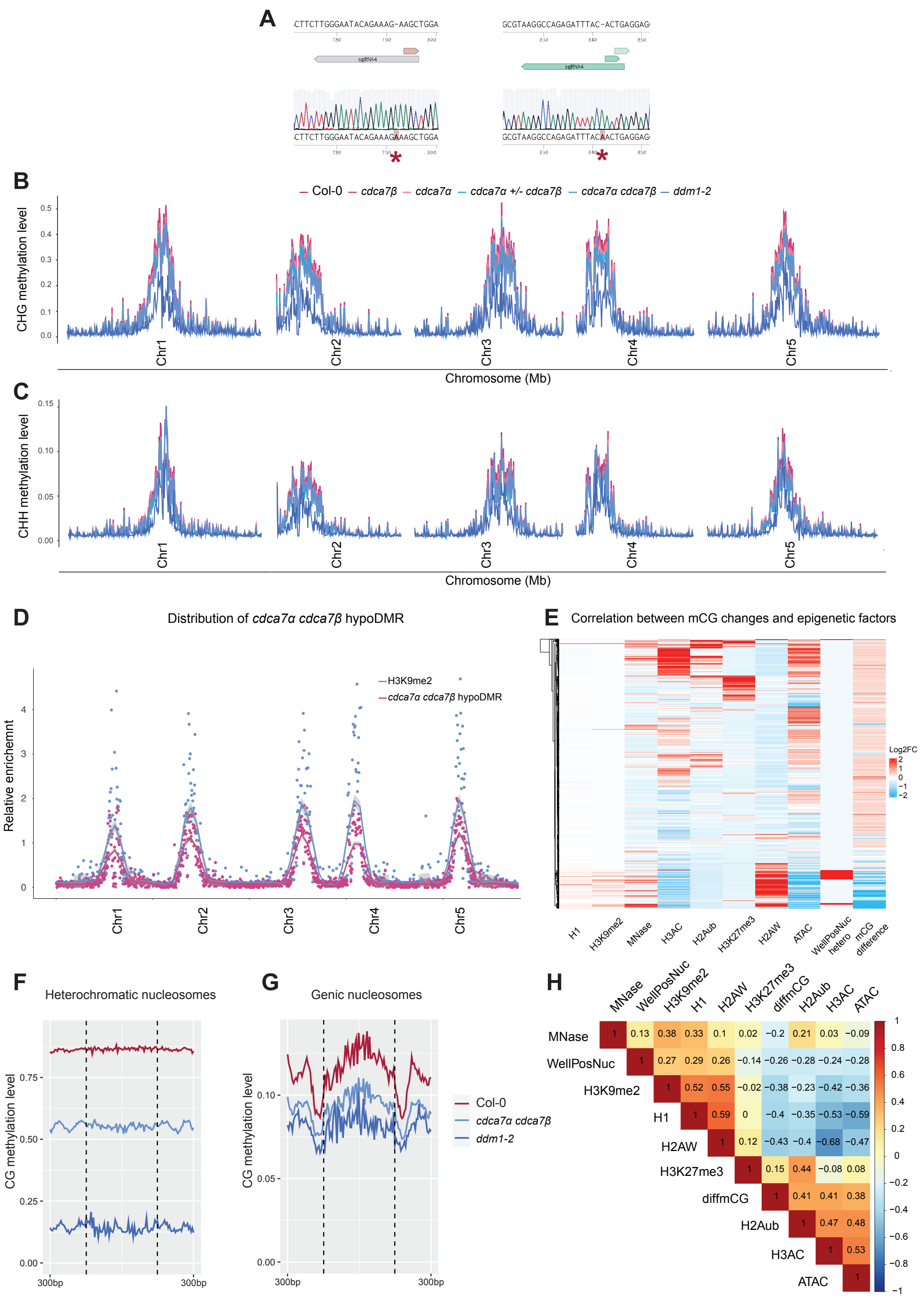

Fig. S2. *CDCA7α* and *CDCA7β* promote DNA methylation at heterochromatic nucleosomes.

A. Sanger sequencing confirmation of CRISPR-Cas9 introduced A insertions at the *CDCA7β* and *CDCA7α* coding regions. **B.** Genome-wide CHG methylation landscape of Col-0, *cdca7α, cdca7β, cdca7α+/- cdca7β, cdca7α cdca7β*, and *ddm1-2*. **C.** Genome-

wide CHH methylation landscape of Col-0, *cdca7α, cdca7β, cdca7α$^{+/-}$ cdca7β, cdca7α cdca7β*, and *ddm1-2*. **D.** Distribution of the *cdca7α cdca7β* hypoDMR. H3K9me2 enrichment marks the location of heterochromatin. **E.** Heatmaps showing the log2 fold change of epigenetic features and CG methylation at the *cdca7α cdca7β* DMR in the *cdca7α cdca7β* mutant compared to wild type. Metaplots showing CG methylation levels at **F.** heterochromatic well-positioned nucleosomes and **G.** genic well-positioned nucleosomes of Col-0, *cdca7α cdca7β*, and *ddm1-2*. **H.** Spearman correlation matrix among epigenetic features and changes in CG methylation level in the *cdca7α cdca7β* mutants.

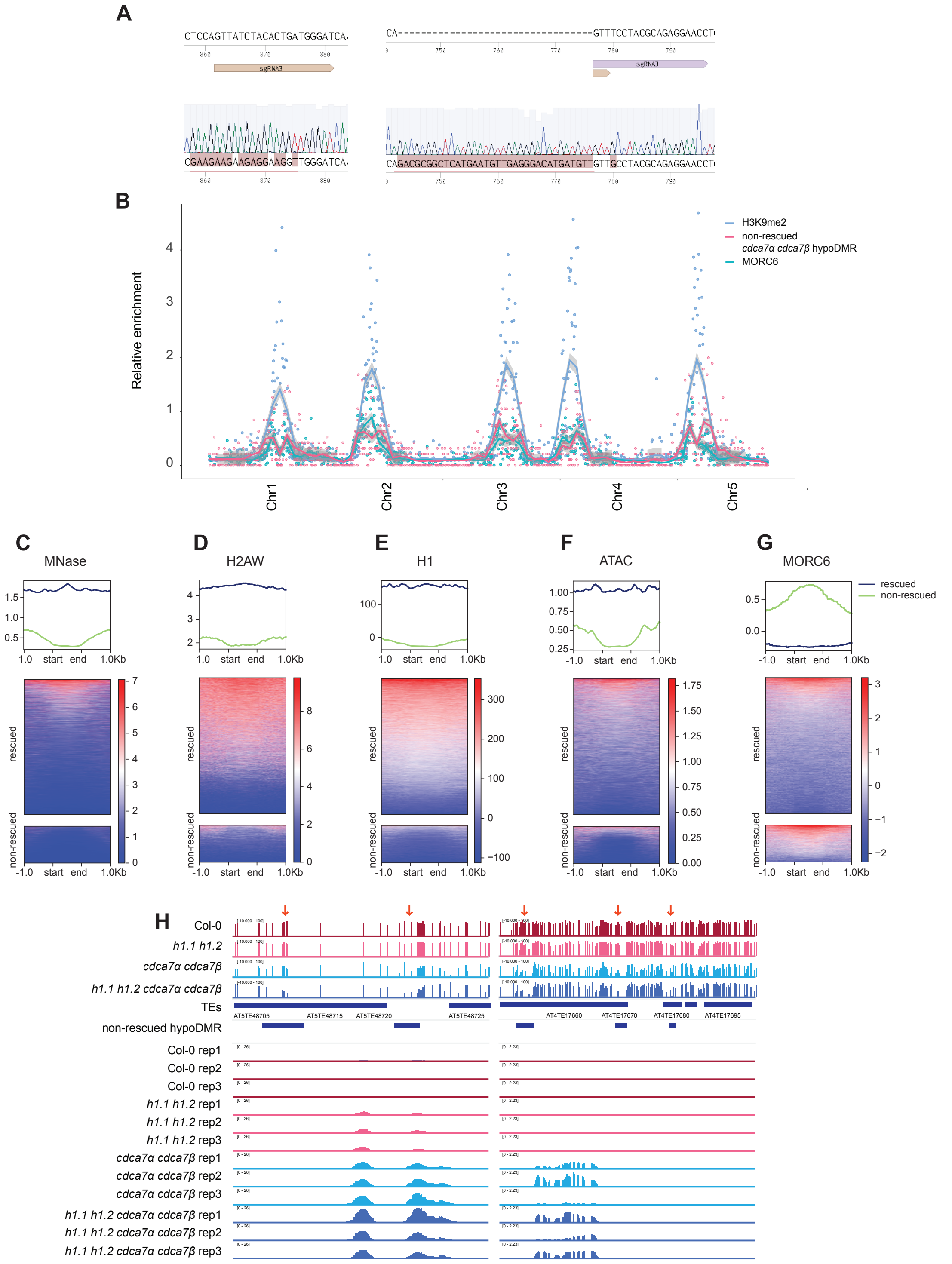

Fig. S3. *CDCA7α* and *CDCA7β* maintain CG methylation independently of H1.

A. Sanger sequencing confirmation of CRISPR-Cas9 introduced mutations at *CDCA7β* and *CDCA7α* coding regions. The red lines indicated the region where mutations were introduced. **B.** Distribution of the non-rescued *cdca7α cdca7β* hypoDMR (regions with CG methylation levels not recovered in *h1.1 h1.2 cdca7α cdca7β*). Relative enrichment of H3K9me2 indicates heterochromatin. Metaplots showing **C.** MNase-seq signal, **D.** H2A.W ChIP-seq signal, **E.** H1

ChIP-seq signal, **F.** ATAC-seq signal, and **G.** MORC6 ChIP-seq signal at non-rescued *cdca7α cdca7β* hypoDMR sites. **H.** Genome browser examples showing CG methylation and gene expression across Col-0, *h1.1 h1.2, cdca7α cdca7β,* and *h1.1 h1.2 cdca7α cdca7β* at non-rescued hypoDMRs in *h1.1 h1.2 cdca7α cdca7β* mutants. The red arrows indicate the locations of the hypoDMRs in the *h1.1 h1.2 cdca7α cdca7β* mutants.

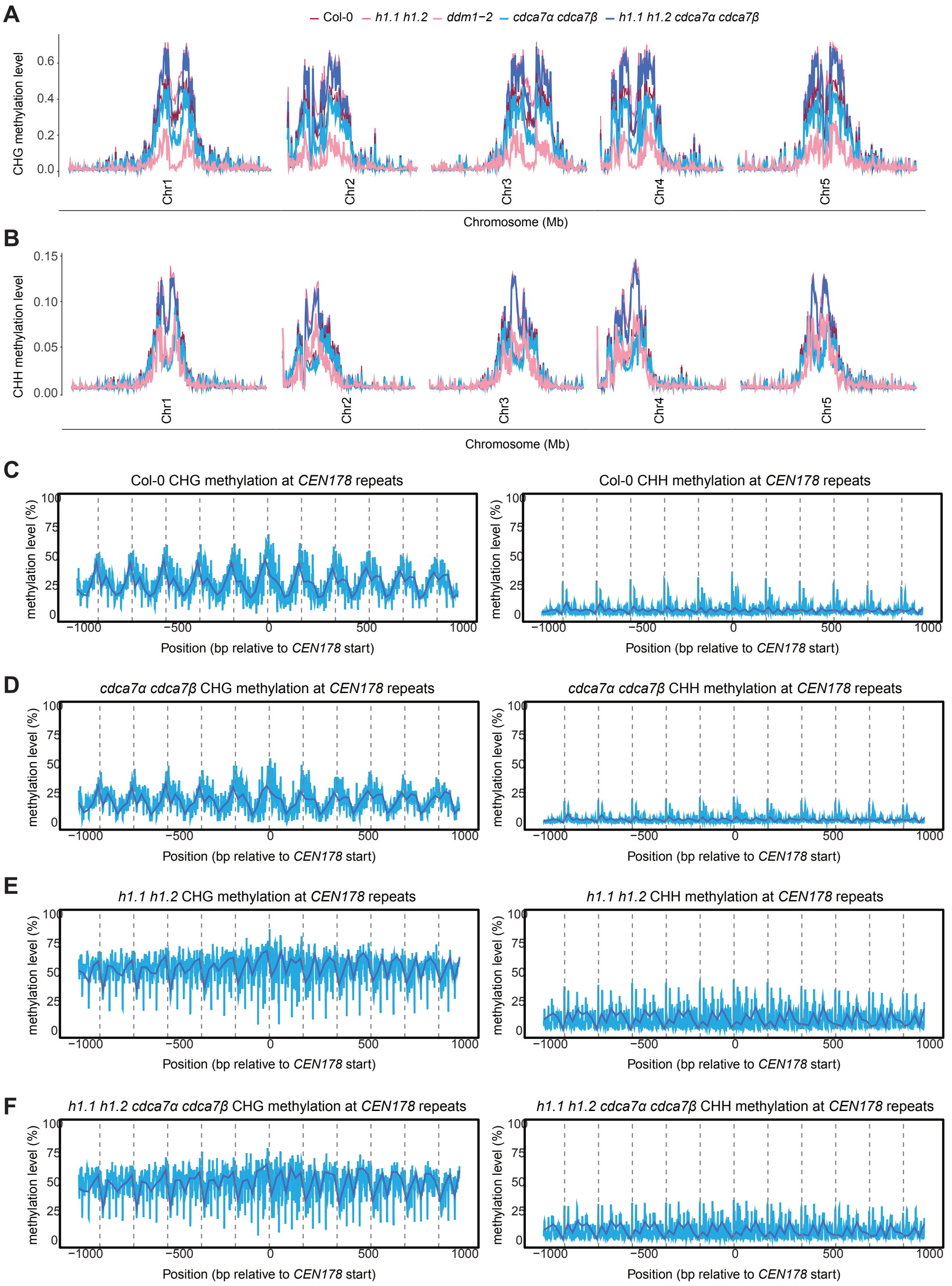

Fig. S4. *CDCA7α* and *CDCA7β* play a minor role in regulating non-CG methylation at centromeric regions.

Genome-wide **A.** CHG methylation, and **B.** CHH methylation landscapes of Col-0, *h1.1 h1.2, cdca7α cdca7β, h1.1 h1.2 cdca7α cdca7β,* and *ddm1-2.* Metaplots showing the non-CG methylation levels at *CEN178* satellite repeats of **C.** Col-0, **D.** *cdca7α cdca7β*, **E.** *h1.1 h1.2,* and **F.** *h1.1 h1.2 cdca7α cdca7β*.

Table S1. Details of the predicted interaction interfaces between *CDCA7β* and DDM1 from AF3.

Supplementary Table 1 CDCA7β DDM1 Interaction Residues					
**Supplementary Table 2: CDCA7β DDM1 interaction Residues**					
**residue_1**	residue_2	distance	chain_1	chain_2	interaction
**A:ARG:35:**	B:ASN:356:	2.42	A	B	Polar
**A:ARG:35:**	B:ASP:355:	4.02	A	B	Contact
**A:ARG:35:**	B:GLN:218:	3.52	A	B	Contact
**A:ARG:35:**	B:GLY:220:	4.2	A	B	Contact
**A:ARG:35:**	B:TRP:217:	2.97	A	B	HBond,Polar
**A:ARG:38:**	B:ASN:219:	4.32	A	B	Contact
**A:ARG:38:**	B:GLN:218:	2.84	A	B	Polar
**A:ARG:38:**	B:GLU:132:	2.76	A	B	Polar
**A:ARG:38:**	B:GLY:128:	4.95	A	B	Contact
**A:ARG:38:**	B:ILE:124:	3.84	A	B	Contact
**A:ARG:38:**	B:ILE:129:	3.34	A	B	Polar
**A:ARG:45:**	B:PHE:116:	3.61	A	B	Contact
**A:ARG:45:**	B:TYR:113:	4.42	A	B	Contact
**A:ARG:547:**	B:LEU:512:	3.27	A	B	Contact
**A:ARG:547:**	B:TYR:511:	3.54	A	B	Contact
**A:ARG:547:**	B:TYR:513:	3.97	A	B	Contact
**A:ARG:547:**	B:TYR:558:	4.31	A	B	Contact
**A:ARG:549:**	B:ARG:569:	4.96	A	B	Contact
**A:ARG:549:**	B:ASP:554:	4.9	A	B	Contact
**A:ARG:549:**	B:ASP:571:	4.13	A	B	Contact
**A:ARG:549:**	B:SER:573:	3.04	A	B	Polar
**A:ARG:550:**	B:ASP:501:	2.94	A	B	Polar
**A:ARG:550:**	B:GLY:505:	3.4	A	B	Contact
**A:ARG:550:**	B:LEU:512:	3.43	A	B	Polar
**A:ARG:550:**	B:PRO:514:	4.63	A	B	Contact
**A:ARG:550:**	B:SER:510:	3.54	A	B	Polar
**A:ARG:550:**	B:TYR:511:	2.81	A	B	Contact
**A:ARG:550:**	B:TYR:513:	3.37	A	B	Contact
**A:ARG:551:**	B:TYR:511:	3.06	A	B	Contact
**A:ASN:42:**	B:ASN:219:	3.23	A	B	Polar
**A:ASN:42:**	B:LEU:117:	3.67	A	B	Contact
**A:ASN:42:**	B:LYS:120:	4.24	A	B	Contact
**A:ASN:42:**	B:PHE:116:	3.45	A	B	Contact
**A:ASN:42:**	B:TYR:113:	3.34	A	B	HBond,Polar
**A:CYS:34:**	B:GLN:218:	4.49	A	B	Contact

1

**A:GLU:32:**	B:LEU:249:	3.17	A	B	Contact
**A:GLU:32:**	B:LYS:328:	2.62	A	B	Polar
**A:GLU:41:**	B:LYS:120:	2.72	A	B	Polar
**A:GLU:41:**	B:PHE:116:	4.3	A	B	Contact
**A:GLU:536:**	B:LEU:512:	3.83	A	B	Contact
**A:GLY:543:**	B:SER:561:	3.72	A	B	Contact
**A:GLY:543:**	B:TYR:513:	4.92	A	B	Contact
**A:GLY:543:**	B:TYR:558:	3.99	A	B	Contact
**A:ILE:39:**	B:ASN:219:	3.8	A	B	Contact
**A:ILE:39:**	B:GLN:218:	4.53	A	B	Contact
**A:ILE:39:**	B:GLY:220:	3.61	A	B	Contact
**A:ILE:542:**	B:ASP:557:	3.03	A	B	Contact
**A:ILE:542:**	B:SER:561:	4.35	A	B	Contact
**A:ILE:542:**	B:VAL:567:	4.43	A	B	Contact
**A:LEU:43:**	B:ASP:382:	4.79	A	B	Contact
**A:LEU:43:**	B:TYR:113:	4.46	A	B	Contact
**A:LEU:49:**	B:GLN:109:	3.52	A	B	Contact
**A:LEU:49:**	B:LEU:112:	4.55	A	B	Contact
**A:LEU:49:**	B:TYR:113:	3.62	A	B	Contact
**A:LEU:51:**	B:GLN:109:	3.44	A	B	Contact
**A:LEU:51:**	B:ILE:383:	3.68	A	B	Contact
**A:LEU:51:**	B:LEU:106:	3.83	A	B	Contact
**A:LEU:51:**	B:THR:110:	3.42	A	B	Contact
**A:LEU:51:**	B:TYR:113:	3.6	A	B	Contact
**A:LEU:52:**	B:ASP:382:	3.44	A	B	Contact
**A:LEU:52:**	B:ILE:383:	4.79	A	B	Contact
**A:LEU:546:**	B:ARG:569:	3.45	A	B	Contact
**A:LEU:546:**	B:ASP:554:	3.4	A	B	Contact
**A:LEU:546:**	B:ASP:557:	3.48	A	B	Contact
**A:LEU:546:**	B:TYR:513:	4.07	A	B	Contact
**A:LEU:546:**	B:TYR:558:	3.6	A	B	Contact
**A:LEU:54:**	B:GLN:109:	3.68	A	B	Contact
**A:LEU:54:**	B:GLU:105:	3.54	A	B	Contact
**A:LEU:54:**	B:LEU:106:	3.7	A	B	Contact
**A:LEU:58:**	B:GLN:99:	3.89	A	B	Contact
**A:LEU:58:**	B:LEU:103:	3.67	A	B	Contact
**A:LEU:58:**	B:LEU:106:	3.18	A	B	Contact
**A:LEU:58:**	B:LYS:102:	3.74	A	B	Contact

**A:LEU:58:**	B:TRP:393:	3.23	A	B	Polar
**A:LYS:57:**	B:LYS:102:	4.04	A	B	Contact
**A:LYS:59:**	B:GLU:389:	3.34	A	B	Contact
**A:LYS:59:**	B:THR:385:	2.92	A	B	Polar
**A:LYS:59:**	B:TRP:393:	4.23	A	B	Contact
**A:MET:46:**	B:ASP:382:	4.54	A	B	Contact
**A:MET:46:**	B:ILE:383:	3.85	A	B	Contact
**A:MET:46:**	B:TYR:113:	3.74	A	B	Contact
**A:PRO:537:**	B:GLU:562:	3.58	A	B	Contact
**A:PRO:537:**	B:TYR:558:	4.1	A	B	Contact
**A:PRO:60:**	B:GLU:389:	4.35	A	B	Contact
**A:PRO:60:**	B:TRP:393:	4.7	A	B	Contact
**A:SER:29:**	B:ASN:247:	4.11	A	B	Contact
**A:SER:541:**	B:SER:561:	4.52	A	B	Contact
**A:SER:55:**	B:ASP:382:	4.52	A	B	Contact
**A:SER:55:**	B:ILE:383:	2.67	A	B	HBond,Polar
**A:SER:55:**	B:LEU:106:	3.87	A	B	Contact
**A:SER:55:**	B:PHE:384:	4.88	A	B	Contact
**A:SER:55:**	B:THR:385:	4.47	A	B	Contact
**A:TYR:31:**	B:ASN:247:	3.97	A	B	Contact
**A:TYR:31:**	B:GLN:218:	2.85	A	B	Polar
**A:TYR:31:**	B:HIS:243:	2.52	A	B	Polar
**A:TYR:31:**	B:ILE:129:	3.51	A	B	Contact
**A:TYR:31:**	B:ILE:214:	3.78	A	B	Contact
**A:TYR:31:**	B:LEU:244:	4.74	A	B	Contact
**A:TYR:31:**	B:LEU:249:	4.2	A	B	Contact
**A:TYR:31:**	B:TRP:217:	3.16	A	B	Contact
**B:ARG:569:**	A:ARG:549:	4.96	B	A	Contact
**B:ARG:569:**	A:LEU:546:	3.45	B	A	Contact
**B:ASN:219:**	A:ARG:38:	4.32	B	A	Contact
**B:ASN:219:**	A:ASN:42:	3.23	B	A	Polar
**B:ASN:219:**	A:ILE:39:	3.8	B	A	Contact
**B:ASN:247:**	A:SER:29:	4.11	B	A	Contact
**B:ASN:247:**	A:TYR:31:	3.97	B	A	Contact
**B:ASN:356:**	A:ARG:35:	2.42	B	A	Polar
**B:ASP:355:**	A:ARG:35:	4.02	B	A	Contact
**B:ASP:382:**	A:LEU:43:	4.79	B	A	Contact
**B:ASP:382:**	A:LEU:52:	3.44	B	A	Contact

**B:ASP:382:**	A:MET:46:	4.54	B	A	Contact
**B:ASP:382:**	A:SER:55:	4.52	B	A	Contact
**B:ASP:501:**	A:ARG:550:	2.94	B	A	Polar
**B:ASP:554:**	A:ARG:549:	4.9	B	A	Contact
**B:ASP:554:**	A:LEU:546:	3.4	B	A	Contact
**B:ASP:557:**	A:ILE:542:	3.03	B	A	Contact
**B:ASP:557:**	A:LEU:546:	3.48	B	A	Contact
**B:ASP:571:**	A:ARG:549:	4.13	B	A	Contact
**B:GLN:109:**	A:LEU:49:	3.52	B	A	Contact
**B:GLN:109:**	A:LEU:51:	3.44	B	A	Contact
**B:GLN:109:**	A:LEU:54:	3.68	B	A	Contact
**B:GLN:218:**	A:ARG:35:	3.52	B	A	Contact
**B:GLN:218:**	A:ARG:38:	2.84	B	A	Polar
**B:GLN:218:**	A:CYS:34:	4.49	B	A	Contact
**B:GLN:218:**	A:ILE:39:	4.53	B	A	Contact
**B:GLN:218:**	A:TYR:31:	2.85	B	A	Polar
**B:GLN:99:**	A:LEU:58:	3.89	B	A	Contact
**B:GLU:105:**	A:LEU:54:	3.54	B	A	Contact
**B:GLU:132:**	A:ARG:38:	2.76	B	A	Polar
**B:GLU:389:**	A:LYS:59:	3.34	B	A	Contact
**B:GLU:389:**	A:PRO:60:	4.35	B	A	Contact
**B:GLU:562:**	A:PRO:537:	3.58	B	A	Contact
**B:GLY:128:**	A:ARG:38:	4.95	B	A	Contact
**B:GLY:220:**	A:ARG:35:	4.2	B	A	Contact
**B:GLY:220:**	A:ILE:39:	3.61	B	A	Contact
**B:GLY:505:**	A:ARG:550:	3.4	B	A	Contact
**B:HIS:243:**	A:TYR:31:	2.52	B	A	Polar
**B:ILE:124:**	A:ARG:38:	3.84	B	A	Contact
**B:ILE:129:**	A:ARG:38:	3.34	B	A	Polar
**B:ILE:129:**	A:TYR:31:	3.51	B	A	Contact
**B:ILE:214:**	A:TYR:31:	3.78	B	A	Contact
**B:ILE:383:**	A:LEU:51:	3.68	B	A	Contact
**B:ILE:383:**	A:LEU:52:	4.79	B	A	Contact
**B:ILE:383:**	A:MET:46:	3.85	B	A	Contact
**B:ILE:383:**	A:SER:55:	2.67	B	A	HBond,Polar
**B:LEU:103:**	A:LEU:58:	3.67	B	A	Contact
**B:LEU:106:**	A:LEU:51:	3.83	B	A	Contact
**B:LEU:106:**	A:LEU:54:	3.7	B	A	Contact

**B:LEU:106:**	A:LEU:58:	3.18	B	A	Contact
**B:LEU:106:**	A:SER:55:	3.87	B	A	Contact
**B:LEU:112:**	A:LEU:49:	4.55	B	A	Contact
**B:LEU:117:**	A:ASN:42:	3.67	B	A	Contact
**B:LEU:244:**	A:TYR:31:	4.74	B	A	Contact
**B:LEU:249:**	A:GLU:32:	3.17	B	A	Contact
**B:LEU:249:**	A:TYR:31:	4.2	B	A	Contact
**B:LEU:512:**	A:ARG:547:	3.27	B	A	Contact
**B:LEU:512:**	A:ARG:550:	3.43	B	A	Polar
**B:LEU:512:**	A:GLU:536:	3.83	B	A	Contact
**B:LYS:102:**	A:LEU:58:	3.74	B	A	Contact
**B:LYS:102:**	A:LYS:57:	4.04	B	A	Contact
**B:LYS:120:**	A:ASN:42:	4.24	B	A	Contact
**B:LYS:120:**	A:GLU:41:	2.72	B	A	Polar
**B:LYS:328:**	A:GLU:32:	2.62	B	A	Polar
**B:PHE:116:**	A:ARG:45:	3.61	B	A	Contact
**B:PHE:116:**	A:ASN:42:	3.45	B	A	Contact
**B:PHE:116:**	A:GLU:41:	4.3	B	A	Contact
**B:PHE:384:**	A:SER:55:	4.88	B	A	Contact
**B:PRO:514:**	A:ARG:550:	4.63	B	A	Contact
**B:SER:510:**	A:ARG:550:	3.54	B	A	Polar
**B:SER:561:**	A:GLY:543:	3.72	B	A	Contact
**B:SER:561:**	A:ILE:542:	4.35	B	A	Contact
**B:SER:561:**	A:SER:541:	4.52	B	A	Contact
**B:SER:573:**	A:ARG:549:	3.04	B	A	Polar
**B:THR:110:**	A:LEU:51:	3.42	B	A	Contact
**B:THR:385:**	A:LYS:59:	2.92	B	A	Polar
**B:THR:385:**	A:SER:55:	4.47	B	A	Contact
**B:TRP:217:**	A:ARG:35:	2.97	B	A	HBond,Polar
**B:TRP:217:**	A:TYR:31:	3.16	B	A	Contact
**B:TRP:393:**	A:LEU:58:	3.23	B	A	Polar
**B:TRP:393:**	A:LYS:59:	4.23	B	A	Contact
**B:TRP:393:**	A:PRO:60:	4.7	B	A	Contact
**B:TYR:113:**	A:ARG:45:	4.42	B	A	Contact
**B:TYR:113:**	A:ASN:42:	3.34	B	A	HBond,Polar
**B:TYR:113:**	A:LEU:43:	4.46	B	A	Contact
**B:TYR:113:**	A:LEU:49:	3.62	B	A	Contact
**B:TYR:113:**	A:LEU:51:	3.6	B	A	Contact

**B:TYR:113:**	A:MET:46:	3.74	B	A	Contact
**B:TYR:511:**	A:ARG:547:	3.54	B	A	Contact
**B:TYR:511:**	A:ARG:550:	2.81	B	A	Contact
**B:TYR:511:**	A:ARG:551:	3.06	B	A	Contact
**B:TYR:513:**	A:ARG:547:	3.97	B	A	Contact
**B:TYR:513:**	A:ARG:550:	3.37	B	A	Contact
**B:TYR:513:**	A:GLY:543:	4.92	B	A	Contact
**B:TYR:513:**	A:LEU:546:	4.07	B	A	Contact
**B:TYR:558:**	A:ARG:547:	4.31	B	A	Contact
**B:TYR:558:**	A:GLY:543:	3.99	B	A	Contact
**B:TYR:558:**	A:LEU:546:	3.6	B	A	Contact
**B:TYR:558:**	A:PRO:537:	4.1	B	A	Contact
**B:VAL:567:**	A:ILE:542:	4.43	B	A	Contact

Table S2. Details of the predicted interaction interfaces between *CDCA7α* and DDM1 from AF3.

Supplementary Table 2 CDCA7α DDM1 Interaction Residues					
**Supplementary Table 2: CDCA7α DDM1 interaction Residues**					
**residue_1**	residue_2	distance	chain_1	chain_2	interaction
**A:ARG:35:**	B:ASN:356:	2.8	A	B	Polar
**A:ARG:35:**	B:ASP:355:	3.94	A	B	Contact
**A:ARG:35:**	B:GLN:218:	3.53	A	B	Contact
**A:ARG:35:**	B:GLY:220:	4.24	A	B	Contact
**A:ARG:35:**	B:TRP:217:	3.03	A	B	HBond,Polar
**A:ARG:38:**	B:ASN:219:	4.19	A	B	Contact
**A:ARG:38:**	B:GLN:218:	3.56	A	B	Polar
**A:ARG:38:**	B:ILE:124:	3.76	A	B	Contact
**A:ARG:38:**	B:ILE:129:	4.47	A	B	Contact
**A:ARG:394:**	B:ASN:540:	4.43	A	B	Contact
**A:ARG:394:**	B:GLU:589:	4.01	A	B	Contact
**A:ARG:394:**	B:LYS:542:	4.51	A	B	Contact
**A:ARG:394:**	B:SER:591:	4.13	A	B	Contact
**A:ARG:394:**	B:SER:592:	4.89	A	B	Contact
**A:ARG:394:**	B:SER:594:	4.29	A	B	Contact
**A:ARG:410:**	B:GLU:562:	2.6	A	B	Polar
**A:ARG:410:**	B:LYS:563:	4.84	A	B	Contact
**A:ARG:410:**	B:SER:561:	3.02	A	B	Polar
**A:ARG:412:**	B:TYR:511:	4.52	A	B	Contact
**A:ARG:413:**	B:LEU:512:	3.06	A	B	Contact
**A:ARG:413:**	B:PRO:514:	4.94	A	B	Contact
**A:ARG:413:**	B:TYR:513:	3.06	A	B	Polar
**A:ARG:413:**	B:TYR:558:	2.92	A	B	Polar
**A:ARG:45:**	B:PHE:116:	3.58	A	B	Contact
**A:ARG:45:**	B:TYR:113:	4.34	A	B	Contact
**A:ASN:42:**	B:ASN:219:	3.21	A	B	Polar
**A:ASN:42:**	B:LEU:117:	3.57	A	B	Contact
**A:ASN:42:**	B:LYS:120:	4.24	A	B	Contact
**A:ASN:42:**	B:PHE:116:	3.42	A	B	Contact
**A:ASN:42:**	B:TYR:113:	3.35	A	B	HBond,Polar
**A:CYS:34:**	B:GLN:218:	4.9	A	B	Contact
**A:GLN:416:**	B:LEU:512:	4.63	A	B	Contact
**A:GLU:32:**	B:LEU:249:	2.51	A	B	Contact
**A:GLU:32:**	B:LYS:328:	2.64	A	B	Polar
**A:GLU:32:**	B:TRP:217:	4.84	A	B	Contact

1

**A:GLU:41:**	B:LYS:120:	2.62	A	B	Polar
**A:GLU:41:**	B:PHE:116:	4.21	A	B	Contact
**A:GLU:61:**	B:GLN:99:	4.28	A	B	Contact
**A:GLU:61:**	B:LYS:102:	2.38	A	B	Polar
**A:GLY:406:**	B:ASP:557:	3.75	A	B	Contact
**A:GLY:406:**	B:SER:561:	3.9	A	B	Contact
**A:GLY:406:**	B:TYR:558:	4.26	A	B	Contact
**A:GLY:407:**	B:SER:561:	3.99	A	B	Contact
**A:ILE:39:**	B:ASN:219:	3.82	A	B	Contact
**A:ILE:39:**	B:GLN:218:	4.7	A	B	Contact
**A:ILE:39:**	B:GLY:220:	3.69	A	B	Contact
**A:ILE:405:**	B:ARG:569:	2.76	A	B	Contact
**A:ILE:405:**	B:ASP:554:	3.42	A	B	Contact
**A:ILE:405:**	B:ASP:557:	3.13	A	B	HBond,Polar
**A:ILE:51:**	B:GLN:109:	3.52	A	B	Contact
**A:ILE:51:**	B:ILE:383:	3.5	A	B	Contact
**A:ILE:51:**	B:LEU:106:	3.69	A	B	Contact
**A:ILE:51:**	B:THR:110:	3.89	A	B	Contact
**A:ILE:51:**	B:TYR:113:	3.49	A	B	Contact
**A:ILE:62:**	B:GLN:99:	4.75	A	B	Contact
**A:ILE:62:**	B:GLU:389:	4.42	A	B	Contact
**A:ILE:62:**	B:SER:392:	3.29	A	B	Contact
**A:ILE:62:**	B:TRP:393:	2.25	A	B	Contact
**A:LEU:251:**	B:ALA:87:	4.04	A	B	Contact
**A:LEU:409:**	B:TYR:511:	3.73	A	B	Contact
**A:LEU:409:**	B:TYR:513:	3.1	A	B	Contact
**A:LEU:409:**	B:TYR:558:	4.42	A	B	Contact
**A:LEU:43:**	B:TYR:113:	4.43	A	B	Contact
**A:LEU:49:**	B:GLN:109:	3.58	A	B	Contact
**A:LEU:49:**	B:LEU:112:	4.41	A	B	Contact
**A:LEU:49:**	B:TYR:113:	3.66	A	B	Contact
**A:LEU:54:**	B:GLN:109:	3.83	A	B	Contact
**A:LEU:54:**	B:GLU:105:	3.72	A	B	Contact
**A:LEU:54:**	B:LEU:106:	3.88	A	B	Contact
**A:LEU:58:**	B:LEU:103:	3.93	A	B	Contact
**A:LEU:58:**	B:LEU:106:	3.84	A	B	Contact
**A:LEU:58:**	B:LYS:102:	3.82	A	B	Contact
**A:LEU:58:**	B:TRP:393:	2.97	A	B	Contact

**A:LYS:247:**	B:GLN:480:	3.61	A	B	Contact
**A:LYS:395:**	B:SER:592:	4.26	A	B	Contact
**A:LYS:59:**	B:ASP:382:	4.21	A	B	Contact
**A:LYS:59:**	B:GLU:389:	3.21	A	B	Contact
**A:LYS:59:**	B:ILE:383:	4.11	A	B	Contact
**A:LYS:59:**	B:THR:385:	2.8	A	B	Polar
**A:MET:396:**	B:GLY:564:	3.71	A	B	Contact
**A:MET:396:**	B:LYS:563:	4.52	A	B	Contact
**A:MET:396:**	B:PHE:565:	3.51	A	B	Contact
**A:MET:46:**	B:ASP:382:	4.98	A	B	Contact
**A:MET:46:**	B:ILE:383:	3.84	A	B	Contact
**A:MET:46:**	B:TYR:113:	3.79	A	B	Contact
**A:MET:52:**	B:ASP:382:	4.13	A	B	Contact
**A:PRO:252:**	B:ALA:87:	4.84	A	B	Contact
**A:PRO:400:**	B:SER:561:	3.87	A	B	Contact
**A:SER:29:**	B:ASN:247:	4.24	A	B	Contact
**A:SER:397:**	B:GLU:566:	2.16	A	B	Polar
**A:SER:397:**	B:GLY:564:	4.33	A	B	Contact
**A:SER:404:**	B:ASP:557:	4.28	A	B	Contact
**A:SER:404:**	B:SER:561:	4.19	A	B	Contact
**A:SER:55:**	B:ASP:382:	4.37	A	B	Contact
**A:SER:55:**	B:ILE:383:	2.85	A	B	HBond,Polar
**A:SER:55:**	B:LEU:106:	4.01	A	B	Contact
**A:TYR:31:**	B:ASN:247:	3.98	A	B	Contact
**A:TYR:31:**	B:GLN:218:	3.11	A	B	Polar
**A:TYR:31:**	B:HIS:243:	2.69	A	B	Polar
**A:TYR:31:**	B:ILE:129:	3.32	A	B	Contact
**A:TYR:31:**	B:ILE:214:	3.67	A	B	Contact
**A:TYR:31:**	B:LEU:244:	4.83	A	B	Contact
**A:TYR:31:**	B:LEU:249:	4.25	A	B	Contact
**A:TYR:31:**	B:TRP:217:	3	A	B	Contact
**A:VAL:140:**	B:VAL:91:	4.04	A	B	Contact
**B:ALA:87:**	A:LEU:251:	4.04	B	A	Contact
**B:ALA:87:**	A:PRO:252:	4.84	B	A	Contact
**B:ARG:569:**	A:ILE:405:	2.76	B	A	Contact
**B:ASN:219:**	A:ARG:38:	4.19	B	A	Contact
**B:ASN:219:**	A:ASN:42:	3.21	B	A	Polar
**B:ASN:219:**	A:ILE:39:	3.82	B	A	Contact

**B:ASN:247:**	A:SER:29:	4.24	B	A	Contact
**B:ASN:247:**	A:TYR:31:	3.98	B	A	Contact
**B:ASN:356:**	A:ARG:35:	2.8	B	A	Polar
**B:ASN:540:**	A:ARG:394:	4.43	B	A	Contact
**B:ASP:355:**	A:ARG:35:	3.94	B	A	Contact
**B:ASP:382:**	A:LYS:59:	4.21	B	A	Contact
**B:ASP:382:**	A:MET:46:	4.98	B	A	Contact
**B:ASP:382:**	A:MET:52:	4.13	B	A	Contact
**B:ASP:382:**	A:SER:55:	4.37	B	A	Contact
**B:ASP:554:**	A:ILE:405:	3.42	B	A	Contact
**B:ASP:557:**	A:GLY:406:	3.75	B	A	Contact
**B:ASP:557:**	A:ILE:405:	3.13	B	A	HBond,Polar
**B:ASP:557:**	A:SER:404:	4.28	B	A	Contact
**B:GLN:109:**	A:ILE:51:	3.52	B	A	Contact
**B:GLN:109:**	A:LEU:49:	3.58	B	A	Contact
**B:GLN:109:**	A:LEU:54:	3.83	B	A	Contact
**B:GLN:218:**	A:ARG:35:	3.53	B	A	Contact
**B:GLN:218:**	A:ARG:38:	3.56	B	A	Polar
**B:GLN:218:**	A:CYS:34:	4.9	B	A	Contact
**B:GLN:218:**	A:ILE:39:	4.7	B	A	Contact
**B:GLN:218:**	A:TYR:31:	3.11	B	A	Polar
**B:GLN:480:**	A:LYS:247:	3.61	B	A	Contact
**B:GLN:99:**	A:GLU:61:	4.28	B	A	Contact
**B:GLN:99:**	A:ILE:62:	4.75	B	A	Contact
**B:GLU:105:**	A:LEU:54:	3.72	B	A	Contact
**B:GLU:389:**	A:ILE:62:	4.42	B	A	Contact
**B:GLU:389:**	A:LYS:59:	3.21	B	A	Contact
**B:GLU:562:**	A:ARG:410:	2.6	B	A	Polar
**B:GLU:566:**	A:SER:397:	2.16	B	A	Polar
**B:GLU:589:**	A:ARG:394:	4.01	B	A	Contact
**B:GLY:220:**	A:ARG:35:	4.24	B	A	Contact
**B:GLY:220:**	A:ILE:39:	3.69	B	A	Contact
**B:GLY:564:**	A:MET:396:	3.71	B	A	Contact
**B:GLY:564:**	A:SER:397:	4.33	B	A	Contact
**B:HIS:243:**	A:TYR:31:	2.69	B	A	Polar
**B:ILE:124:**	A:ARG:38:	3.76	B	A	Contact
**B:ILE:129:**	A:ARG:38:	4.47	B	A	Contact
**B:ILE:129:**	A:TYR:31:	3.32	B	A	Contact

**B:ILE:214:**	A:TYR:31:	3.67	B	A	Contact
**B:ILE:383:**	A:ILE:51:	3.5	B	A	Contact
**B:ILE:383:**	A:LYS:59:	4.11	B	A	Contact
**B:ILE:383:**	A:MET:46:	3.84	B	A	Contact
**B:ILE:383:**	A:SER:55:	2.85	B	A	HBond,Polar
**B:LEU:103:**	A:LEU:58:	3.93	B	A	Contact
**B:LEU:106:**	A:ILE:51:	3.69	B	A	Contact
**B:LEU:106:**	A:LEU:54:	3.88	B	A	Contact
**B:LEU:106:**	A:LEU:58:	3.84	B	A	Contact
**B:LEU:106:**	A:SER:55:	4.01	B	A	Contact
**B:LEU:112:**	A:LEU:49:	4.41	B	A	Contact
**B:LEU:117:**	A:ASN:42:	3.57	B	A	Contact
**B:LEU:244:**	A:TYR:31:	4.83	B	A	Contact
**B:LEU:249:**	A:GLU:32:	2.51	B	A	Contact
**B:LEU:249:**	A:TYR:31:	4.25	B	A	Contact
**B:LEU:512:**	A:ARG:413:	3.06	B	A	Contact
**B:LEU:512:**	A:GLN:416:	4.63	B	A	Contact
**B:LYS:102:**	A:GLU:61:	2.38	B	A	Polar
**B:LYS:102:**	A:LEU:58:	3.82	B	A	Contact
**B:LYS:120:**	A:ASN:42:	4.24	B	A	Contact
**B:LYS:120:**	A:GLU:41:	2.62	B	A	Polar
**B:LYS:328:**	A:GLU:32:	2.64	B	A	Polar
**B:LYS:542:**	A:ARG:394:	4.51	B	A	Contact
**B:LYS:563:**	A:ARG:410:	4.84	B	A	Contact
**B:LYS:563:**	A:MET:396:	4.52	B	A	Contact
**B:PHE:116:**	A:ARG:45:	3.58	B	A	Contact
**B:PHE:116:**	A:ASN:42:	3.42	B	A	Contact
**B:PHE:116:**	A:GLU:41:	4.21	B	A	Contact
**B:PHE:565:**	A:MET:396:	3.51	B	A	Contact
**B:PRO:514:**	A:ARG:413:	4.94	B	A	Contact
**B:SER:392:**	A:ILE:62:	3.29	B	A	Contact
**B:SER:561:**	A:ARG:410:	3.02	B	A	Polar
**B:SER:561:**	A:GLY:406:	3.9	B	A	Contact
**B:SER:561:**	A:GLY:407:	3.99	B	A	Contact
**B:SER:561:**	A:PRO:400:	3.87	B	A	Contact
**B:SER:561:**	A:SER:404:	4.19	B	A	Contact
**B:SER:591:**	A:ARG:394:	4.13	B	A	Contact
**B:SER:592:**	A:ARG:394:	4.89	B	A	Contact

**B:SER:592:**	A:LYS:395:	4.26	B	A	Contact
**B:SER:594:**	A:ARG:394:	4.29	B	A	Contact
**B:THR:110:**	A:ILE:51:	3.89	B	A	Contact
**B:THR:385:**	A:LYS:59:	2.8	B	A	Polar
**B:TRP:217:**	A:ARG:35:	3.03	B	A	HBond,Polar
**B:TRP:217:**	A:GLU:32:	4.84	B	A	Contact
**B:TRP:217:**	A:TYR:31:	3	B	A	Contact
**B:TRP:393:**	A:ILE:62:	2.25	B	A	Contact
**B:TRP:393:**	A:LEU:58:	2.97	B	A	Contact
**B:TYR:113:**	A:ARG:45:	4.34	B	A	Contact
**B:TYR:113:**	A:ASN:42:	3.35	B	A	HBond,Polar
**B:TYR:113:**	A:ILE:51:	3.49	B	A	Contact
**B:TYR:113:**	A:LEU:43:	4.43	B	A	Contact
**B:TYR:113:**	A:LEU:49:	3.66	B	A	Contact
**B:TYR:113:**	A:MET:46:	3.79	B	A	Contact
**B:TYR:511:**	A:ARG:412:	4.52	B	A	Contact
**B:TYR:511:**	A:LEU:409:	3.73	B	A	Contact
**B:TYR:513:**	A:ARG:413:	3.06	B	A	Polar
**B:TYR:513:**	A:LEU:409:	3.1	B	A	Contact
**B:TYR:558:**	A:ARG:413:	2.92	B	A	Polar
**B:TYR:558:**	A:GLY:406:	4.26	B	A	Contact
**B:TYR:558:**	A:LEU:409:	4.42	B	A	Contact
**B:VAL:91:**	A:VAL:140:	4.04	B	A	Contact
